# Surveillance of the efficacy of artemether-lumefantrine and artesunate-amodiaquine for the treatment of uncomplicated *Plasmodium falciparum* among children under five in Togo, 2005-2009

**DOI:** 10.1186/1475-2875-11-338

**Published:** 2012-10-08

**Authors:** Monique A Dorkenoo, Amy Barrette, Yao M Agbo, Hervé Bogreau, Séenam Kutoati, Yao K Sodahlon, Kodjo Morgah

**Affiliations:** 1 Faculté Mixte de Médecine et de Pharmacie, Université de Lomé, Lomé, BP1515, Togo; 2 Global Malaria Programme, World Health Organization, 20 Avenue Appia, Geneva 27, 1211, Switzerland; 3 Unité de Recherche en Biologie et Epidémiologie Parasitaires, Institut de Médecine Tropicale des Services de Santé de l’Armée, Allée du Médecin, colonnel Jamot, Parc du PHARO, Marseille cedex 07, BP 60109, 13262, France; 4 Service de Médecine Polyvalente-Urgences Médicales du CHU Amiens Picardie, Faculté de Médecine de l’Université Jules Vernes de Picardie, 37 Rue Alfred Lemaire, Amiens, 80000, France; 5 Mectizan Donation Programme, 325, Swaton way, Decatur, GA, 30030, USA; 6 Ministère de la Santé, Angle avenue, Sarakawa et avenue 24 janvier, Lomé, BP 336, Togo

**Keywords:** Malaria, *Plasmodium falciparum*, Therapeutic efficacy, Artemisinin-based combination, Togo

## Abstract

**Background:**

Malaria remains a major public health problem in Togo. The national malaria control programme in Togo changed the anti-malarial treatment policy from monotherapy to artemisinin combination therapy in 2004. This study reports the results of therapeutic efficacy studies conducted on artemether-lumefantrine and artesunate-amodiaquine for the treatment of uncomplicated *Plasmodium falciparum* malaria in Togo, between 2005 and 2009.

**Methods:**

Children between 6 and 59 months of age, who were symptomatically infected with *P. falciparum,* were treated with either artemether-lumefantrine or artesunate-amodiaquine. The primary end-point was the 28-day cure rate, PCR-corrected for reinfection and recrudescence. Studies were conducted according to the standardized WHO protocol for the assessment of the efficacy of anti-malarial treatment. Differences between categorical data were compared using the chi-square test or the Fisher’s exact test where cell counts were ≤ 5. Differences in continuous data were compared using a *t*-test.

**Results:**

A total of 16 studies were conducted in five sentinel sites, with 459, 505 and 332 children included in 2005, 2007 and 2009, respectively. The PCR-corrected 28-day cure rates using the per-protocol analysis were between 96%-100% for artemether-lumefantrine and 94%-100% for artesunate-amodiaquine.

**Conclusions:**

Both formulations of artemisinin-based combination therapy were effective over time and no severe adverse events related to the treatment were reported during the studies.

## Background

Malaria remains a major public health problem in Togo, an area of high transmission, with prevalence highest during the rainy season. Children carry the highest burden: in 2009, 56% of all reported cases and 73% of all reported deaths occurred among children under five years of age [1]. In 2001, following an increase in *Plasmodium falciparum* resistance to anti-malarial medicines, the Togo National Malaria Control Programme (NMCP) set up a routine surveillance system, with five sentinel sites covering the distinct epidemiological zones of the country. Between 2001 and 2003, treatment failures with chloroquine and sulphadoxine−pyrimethamine reached 63% and 26%, respectively [2]. A national consensus meeting in May 2004 led to the adoption of two different forms of artemisinin-based combination therapy (ACT) for the treatment of uncomplicated malaria in Togo: artemether-lumefantrine and artesunate-amodiaquine. The NMCP began to monitor the therapeutic efficacy of these two combinations in 2005. This article reports the efficacy of both forms of ACT for the treatment of uncomplicated malaria between 2005 and 2009.

## Methods

### Sentinel sites

The studies were conducted in the following sites: 1) Agbalpédogan Jérusalem Medical Centre and Adakpamé District Hospital in the capital city of Lomé; 2) La Providence Medical Centre in Kouvé; 3) Sodoké and Kpangalam “Bon Secours” Medical Centres located in Sodoké; 4) Doufelgou District Hospital in Niamtougou; and 5) Tantigou “Yendoubé” Paediatric Hospital in Dapaong. The last four sentinel sites extend north from Lomé, in the order listed above, by 92 km, 350 km, 425 km and 620 km, respectively. Studies were conducted during the high transmission season for malaria, between October and December, except for Lomé, where the study was conducted from August to November.

### Study design

The studies were based on the standardized World Health Organization (WHO) protocol for the assessment of the efficacy of anti-malarial treatment [3]. Patients who presented for care at one of the health centres were eligible for inclusion if they met the following criteria: age between 6 and 59 months; fever (≥ 37.5 °C); *P. falciparum* mono-infection with parasite density between 2,000 and 200,000 asexual parasites/mm^3^. Exclusion criteria included: one or more signs of severe or complicated malaria, mixed infection or infection with another species, malnutrition, concomitant disease, chronic or severe diseases, hypersensitivity or contra-indication to the study drugs and absence of informed consent of the parents. Clinical examination, including measurement of axillary temperature and blood smear for parasite counts, was performed at enrolment and on day 1, 2, 3, 7, 14, 21 and 28. Parasite counts were determined on Giemsa-stained thick films and recorded as the number of parasites per 200 white blood cells at admission and per 1,000 white cells on follow-up days based on a putative count of 6,000 white blood cells per microlitre of blood. The presence of gametocytes was also recorded in the 2007 and 2009 trials. Changes to haemoglobin levels after ACT treatment in 2007 and 2009 were measured using Hemocue haemoglobinometer. Capillary blood was sampled for haemoglobin at day 0, day 14 and day 28. All studies were approved by the Bioethics committee of the Ministry of Health and WHO Ethical Research Committee in 2007. Sample size was calculated according to WHO recommendations [4]. The sample size was estimated with a treatment success of 95%, the minimum expected efficacy of an ACT in a region where it has never been used. The confidence level was estimated at 95% and a precision level of 10%, for a target sample size of 50 children per treatment arm and per site. An additional 20% was added to ensure the sample size would be achieved after patients were excluded due to loss to follow-up and withdrawals.

### Study medicines

Artemether-lumefantrine (Novartis Pharma, Switzerland) tablets containing 20 mg of artemether and 120 mg lumefantrine were administered every 12 hours over 3 days. Treatment was given without co-administration of fatty food. Weight-based dosing was applied, with one tablet for children weighing 5-14 kg and two tablets for children weighing 15-24 kg. Artesunate and amodiaquine were administered at an average dose of 4 mg/kg/day and 10 mg/kg/day over 3 days, respectively. Two different presentations of artesunate-amodiaquine were used: in 2005 and 2007, artesunate was purchased from Sanofi Synthélabo (France), and amodiaquine was supplied by Hoechst Marion Roussel (France). A co-blister of artesunate-amodiaquine manufactured by sanofi aventis (France) was used in 2009. The medicines were administered under supervision and allocated randomly. The purpose of the studies was not to compare the efficacy of the two combinations, but to monitor their efficacy independently.

### Outcome assessment

Treatment outcomes were classified based on an assessment of parasitological and clinical outcomes, according to the methods recommended by WHO in 2003 [3], with modifications suggested by WHO in 2005 [5]. Unlike the 2003 protocol, which limits late treatment failures to patients with parasites on day 28, the 2005 modification considers a patient to have failed treatment when parasites are detected on any day between day 7 and day 28. In these studies, therapeutic response was classified on day 28 as either: early treatment failure (ETF), late clinical failure (LCF), late parasitological failure (LPF), or adequate clinical and parasitological response (ACPR). All treatment failures (TF = ETF + LCF + LPF) were treated with quinine at the day of failure. Children who developed severe malaria during the follow-up were treated with parenteral quinine. The proportion of cases still positive on day 3 was also recorded. PCR was conducted in order to distinguish between recrudescence and reinfection. In 2005, only two molecular markers for PCR (*msp1* and *msp2)* were used. In 2007 and 2009, all three molecular markers (*msp1, msp2* and *glurp*) were used. Parasite DNA was extracted from blood spots collected on day 0 and on the day of reappearance of asexual parasitaemia [6]. Patients who were lost to follow-up, had protocol violations, whose treatment failure was due to reinfection, or whose type of treatment failure could not be determined through PCR were excluded from the per-protocol analysis, as recommended in the WHO protocol [3].

### Statistical analysis

All data were entered twice in the WHO Microsoft® Office Excel spreadsheet. Analysis was conducted with Stata/IC 11.0 (Stata Corporation, College Station, Texas 77845 USA). Differences between categorical data were compared using the chi-square test or the Fisher’s exact test where cell counts were ≤ 5. Differences in continuous data were compared using a *t*-test. Exact 95% confidence intervals were calculated for the treatment failure rates.

## Results

Patients treated with artesunate-amodiaquine (n = 651) had a sex ratio of 1.1, a mean age of 2.7 years (standard deviation (SD) = 1.3 years), a mean weight of 12.2 kg (SD = 3.1 kg), and a mean temperature of 38.7 °C (SD = 1.0 °C). The geometric mean parasitaemia on day 0 was 23,494/μl (95% CI: 21,376-25,822). Patients treated with artemether-lumefantrine (n = 645) had a sex ratio of 1.3, a mean age of 2.9 years (SD = 1.3 years), a mean weight of 12.5 kg (SD = 3.1 kg), a mean temperature of 38.7 °C (SD = 1.0 °C). The geometric mean parasitaemia on day 0 was 21,183/μl (95% CI: 19,336-23,206).

Differences in patient characteristics on admission were investigated among studies conducted at the same site and on the same treatment between 2005 and 2007 (Niamtougou), and 2005 and 2009 (Dapaong and Kouvé) (Table 1). Studies in Lomé and Sokodé were conducted in 2007 only. In the Kouvé studies of artesunate-amodiaquine, there was a higher proportion of males in 2005 (61%) than in 2009 (46%) (P = 0.04). In Niamtougou, the mean weight of patients was higher in 2005 (13.8 kg) than in 2007 (11.9 kg) (P < 0.001). No other differences were observed in admission characteristics over time.

**Table 1:** **Patient characteristics at the time of admission for treatment of *P. falciparum *with artemether-lumefantrine (AL) and artesunate-amodiaquine (ASAQ), by sentinel site and by year (2005 to 2009)**

**drug**	**site**	**district**	**province**	**year**	**n**	**male/female**	**mean age years (SD)**		**mean weight kg (SD)**		**mean temp °C (SD)**		**geometric mean parasitaemia (/ul)**	
AL	Dapaong	Tone	Savanes	2005	75	38/37	3.4	(1.5)	13.3	(3.5)	38.8	(0.8)	22037	(17390-27925)
AL	Dapaong	Tone	Savanes	2009	82	45/37	3.0	(1.3)	12.2	(3.1)	38.5	(0.6)	34509	(26784-44464)
AL	Kouvé	Yoto	Maritime	2005	78	43/35	2.6	(1.3)	11.7	(3.2)	38.6	(1.0)	16866	(13011-21863)
AL	Kouvé	Yoto	Maritime	2009	84	50/34	2.6	(1.2)	11.7	(2.9)	38.8	(0.9)	22043	(16877-28791)
AL	Lomé	Lomé	Maritime	2007	86	51/35	3.1	(1.4)	13.1	(3.1)	39.2	(0.9)	24058	(18724-30911)
AL	Niamtougou	Doufelgou	Kara	2005	76	42/34	3.4	(1.3)	13.8	(3.1)	38.4	(0.8)	13074	(9822-17403)
AL	Niamtougou	Doufelgou	Kara	2007	82	47/35	2.8	(1.2)	11.9	(2.7)	38.6	(1.0)	19430	(15209-24823)
AL	Sokodé	Tchaoudjo	Centrale	2007	82	48/34	2.9	(1.3)	12.3	(2.9)	38.4	(0.8)	21677	(16947-27727)
ASAQ	Dapaong	Tone	Savanes	2005	75	34/41	3.2	(1.4)	12.7	(3.1)	38.5	(0.8)	27339	(21628-34559)
ASAQ	Dapaong	Tone	Savanes	2009	81	42/39	3.1	(1.4)	12.6	(3.0)	38.6	(0.8)	36599	(28729-46624)
ASAQ	Kouvé	Yoto	Maritime	2005	78	48/30	2.3	(1.3)	11.2	(3.0)	38.7	(1.1)	16004	(11861-21596)
ASAQ	Kouvé	Yoto	Maritime	2009	85	39/46	2.3	(1.1)	11.1	(2.8)	38.9	(1.0)	21346	(16300-27954)
ASAQ	Lomé	Lomé	Maritime	2007	85	47/38	2.9	(1.3)	12.5	(2.9)	39.0	(1.0)	26065	(19569-34717)
ASAQ	Niamtougou	Doufelgou	Kara	2005	77	37/40	2.8	(1.5)	12.6	(3.9)	38.3	(0.9)	15893	(11593-21789)
ASAQ	Niamtougou	Doufelgou	Kara	2007	83	51/32	2.9	(1.2)	11.9	(2.8)	38.6	(1.0)	18118	(14330-22906)
ASAQ	Sokodé	Tchaoudjo	Centrale	2007	87	49/38	3.1	(1.2)	13.1	(3.1)	38.7	(0.9)	35226	(28287-43865)

The overall PCR-corrected treatment failure rates remained low: between 0-4.4% for artemether-lumefantrine, and 0-6% for artesunate-amodiaquine (Table 2). Both treatments resulted in a rapid clearance of the parasites. No ETFs were reported. Among the 1296 patients included in the 16 studies, 93% cleared their parasitaemia by day 2 and 98.4% by day 3. Patients still positive on day 2 or day 3 presented with very low parasitaemia, usually less than 50 parasites/μl. The proportion of patients positive on day 3 was 2.2% in 2005, 1.6% in 2007, and 0.6% in 2009.

**Table 2:** **Parasitological and clinical outcomes among patients treated for *P. falciparum *malaria with artemether-lumefantrine (AL) and artesunate-amodiaquine (ASAQ), by year and site (2005-2009)**

				**Non-PCR-corrected**							**PCR-corrected**							
**Drug**	**Site**	**Year**	**n**	**excl/loss**	**ACPR**	**LCF**	**LPF**	**TF**	**(%)**	**(Exact 95% CI)**	**excl/loss**	**ACPR**	**LCF**	**LPF**	**TF**	**(%)**	**(Exact 95% CI)**	**Kaplan-Meier**
AL	Dapaong	2005	75	1	71	3	0	3	(4.1)	(0.8-11.4)	4	71	0	0	0	(0.0)	(0.0-5.1)	0.0 (0.0-0.0)
AL	Dapaong	2009	82	0	73	2	7	9	(11.0)	(5.1-19.8)	9	73	0	0	0	(0.0)	(0.0-4.9)	0.0 (0.0-0.0)
AL	Kouvé	2005	78	3	65	4	6	10	(13.3)	(6.6-23.2)	10	65	0	3	3	(4.4)	(0.9-12.4)	4.1 (1.3-12.2)
AL	Kouvé	2009	84	0	68	2	14	16	(19.1)	(11.3-29.1)	16	68	0	0	0	(0.0)	(0.0-5.3)	0.0 (0.0-0.0)
AL	Lomé	2007	86	7	72	4	3	7	(8.9)	(3.6-17.4)	13	72	1	0	1	(1.4)	(0.0-7.4)	1.3 (0.2-8.8)
AL	Niamtougou	2005	76	0	67	1	8	9	(11.8)	(5.6-21.3)	7	67	1	1	2	(2.9)	(0.4-10.1)	2.7 (0.7-10.5)
AL	Niamtougou	2007	82	1	56	10	15	25	(30.9)	(21.1-42.1)	26	56	0	0	0	(0.0)	(0.0-6.4)	0.0 (0.0-0.0)
AL	Sokodé	2007	82	6	66	2	8	10	(13.2)	(6.5-22.9)	15	66	0	1	1	(1.5)	(0.0-8.0)	1.4 (0.2-9.7)
AL	Overall		645	18	538	28	61	89	(14.2)	(11.6-17.2)	100	538	2	5	7	(1.3)	(0.3-4.1)	1.2 (0.5-26.2)
ASAQ	Dapaong	2005	75	0	74	0	1	1	(1.3)	(0.0-7.2)	1	74	0	0	0	(0.0)	(0.0-4.9)	0.0 (0.0-0.0)
ASAQ	Dapaong	2009	81	0	74	1	6	7	(8.6)	(3.5-17.0)	6	74	1	0	1	(1.3)	(0.0-7.2)	1.3 (0.2-8.8)
ASAQ	Kouvé	2005	78	3	72	0	3	3	(4.0)	(0.8-11.4)	6	72	0	0	0	(0.0)	(0.0-5.1)	0.0 (0.0-0.0)
ASAQ	Kouvé	2009	85	1	78	2	4	6	(7.1)	(2.7-14.9)	7	78	0	0	0	(0.0)	(0.0-4.6)	0.0 (0.0-0.0)
ASAQ	Lomé	2007	85	3	79	1	2	3	(3.7)	(0.8-10.3)	6	79	0	0	0	(0.0)	(0.0-4.6)	0.0 (0.0-0.0)
ASAQ	Niamtougou	2005	77	2	63	1	11	12	(16.0)	(8.6-26.3)	10	63	0	4	4	(6.0)	(1.7-14.6)	5.6 (2.1-14.2)
ASAQ	Niamtougou	2007	83	6	66	3	8	11	(14.3)	(7.4-24.1)	16	66	0	1	1	(1.5)	(0.0-8.0)	1.3 (0.2-9.1)
ASAQ	Sokodé	2007	87	4	74	1	8	9	(10.8)	(5.1-19.6)	13	74	0	0	0	(0.0)	(0.0-4.9)	0.0 (0.0-0.0)
ASAQ	Overall		651	19	580	9	43	52	(8.2)	(6.2-10.6)	65	580	1	5	6	(1.0)	(0.3-3.5)	1.0 (0.3-2.2)

*ACPR*: adequate clinical and parasitological response; *LCF*: late clinical failure; *LPF*: late parasitological failure; *TF*: treatment failures (TF = LCF + LPF).

Non-PCR-corrected treatment failure rates among studies conducted in 2005, 2007 and 2009 were 8.4%, 13.6% and 11.5%, respectively. However, the proportion of non-PCR-corrected treatment failures subsequently PCR-corrected as recrudescence decreased from 23.7% in 2005, to 4.6% in 2007 and 2.6% in 2009. There was a corresponding increase in the proportion classified as reinfections by PCR, from 55.3% in 2005, to 80% in 2007 and 84.2% in 2009 (P = 0.004). This increase was observed in all three sites where studies were conducted twice within the four-year period, but the increase was most pronounced in Kouvé, with 28.6% in 2005 and 71.4% in 2009 (P = 0.04), and in Niamtougou, where the proportion of non-PCR-corrected treatment failures subsequently PCR-corrected as reinfections increased from 24.4% in 2005 to 75.6% in 2007 (P = 0.001). The increase was consistent in both treatment groups. No severe adverse events related to the ACT were reported during the 16 studies.

Haemoglobin levels for subjects in both treatment groups increased progressively from day 0 to day 14 and to day 28 (Table 3). Significant differences in haemoglobin levels were observed on day 14 and day 28 when compared to day 0 for all sites and treatments (P < 0.05), except the artemether-lumefantrine studies conducted in Lomé and Niamtougou in 2007 and both forms of ACT in Dapong in 2009, where the mean increase was less than 0.3 g/dl.

**Table 3:** Mean (standard deviation) hemoglobin levels at day 0, day 14 and day 28, following treatment with artemether-lumefantrine (AL), artesunate-amodiaquine (ASAQ), in 2007 and 2009

	**2007**			**2009**		
	**D0**	**D14**	**D28**	**D0**	**D14**	**D28**
AL	9.8 (1.8)	10.2 (1.5)	10.9 (1.5)	9.2 (1.6)	9.8 (1.3)	10.5 (1.3)
ASAQ	9.3 (1.8)	10.1 (1.5)	11.0 (1.4)	9.2 (1.7)	9.8 (1.3)	10.9 (1.3)

While there was some variation in the proportion of patients with gametocytes on day 0, no differences were found among sites or treatment regimens at other time points (Table 4). There was a rapid reduction in gametocytes, and no new gametocytes appeared over the 28-day period. No data on gametocytes were available from the studies conducted in 2005.

**Table 4:** Patients with gametocytes on admission and following treatment with artemether-lumefantrine (AL) artesunate-amodiaquine (ASAQ), by site and year (2007 to 2009)

**Drug**	**Site**	**District**	**Province**	**Year**	**Gametocytes**				
					**day 0**	**day 7**	**day 14**	**day 21**	**day 28**
AL	Dapaong	Tone	Savanes	2009	0/82	0/82	0/82	0/80	0/76
AL	Kouvé	Yoto	Maritime	2009	8/84	3/84	1/84	1/84	1/83
AL	Lomé	Lomé	Maritime	2007	0/85	1/81	0/79	0/79	0/76
AL	Niamtougou	Doufelgou	Maritime	2007	9/82	3/81	1/81	0/79	0/71
AL	Sokodé	Tchaoudjo	Centrale	2007	2/82	0/76	0/76	0/76	0/73
ASAQ	Dapaong	Tone	Savanes	2009	0/81	0/81	0/81	0/79	0/76
ASAQ	Kouvé	Yoto	Maritime	2009	7/85	2/85	0/85	0/84	0/84
ASAQ	Lomé	Lomé	Maritime	2007	6/84	3/82	0/82	1/82	0/82
ASAQ	Niamtougou	Doufelgou	Maritime	2007	11/83	7/78	7/79	2/77	0/76
ASAQ	Sokodé	Tchaoudjo	Centrale	2007	5/87	5/83	6/83	2/82	1/81

## Discussion

These studies showed the high therapeutic efficacy of artemether-lumefantrine and artesunate-amodiaquine in Togo between 2005 and 2009. The studies also demonstrated the significant improvement of haemoglobin levels following treatment. In addition, the artemisinin’s gametocytocidal effect was observed by the initial, rapid reduction in gametocytes, and the failure of any new gametocytes to appear over the 28-day period.

In Togo, the efficacy of artemether-lumefantrine was high and did not change between 2005 and 2009, despite the absence of co-administration of fat. This is consistent with a review of studies from sub-Saharan Africa, where it was found that the fat content of standard meals or breast milk was adequate for absorption of lumefantrine [7]. In 2009, artemether-lumefantrine was the first-line treatment in 29 African countries [8]. In a review of 140 studies of the therapeutic efficacy of artemether-lumefantrine in Africa [8], a PCR-corrected treatment failure rate of higher than 10% was observed in only two studies: 13.8% in Ghana and 12.3% in Burkina Faso [9].

Similarly, the efficacy of artesunate-amodiaquine was high in Togo, and did not change between 2005 and 2009. Two different presentations were used in these studies: loose tablets were used in 2005 and 2007, and co-blistered treatment was used in 2009. Despite different presentations, the treatment outcome did not vary significantly over time. In 2009, artesunate-amodiaquine was the first-line treatment for uncomplicated malaria in 22 African countries [8]. However, the 28-day therapeutic efficacy of artesunate-amodiaquine varies substantially across the African continent [8,10] due to the pre-existing resistance of amodiaquine, which also varies across the continent [5]. In a 2003 study in Togo, amodiaquine was observed to be over 90% effective, although follow-up was only 14 days [2].

The studies presented here showed an increase in the proportion of reinfections detected in 2007 and 2009 when compared to 2005. The increase in the proportion of reinfections was likely caused by the addition of a third molecular marker, *glurp*, which improved the ability to distinguish between reinfection and recrudescence. In 2005, only *msp1* and *msp 2* were used. When *glurp* was added in 2007 and 2009, the molecular marker detected 53% and 65% of the reinfections, respectively, demonstrating its high ability to discriminate between a reinfection and a recrudescence. Failure to use all three molecular markers in PCR analyses may result in incorrect conclusions regarding the true efficacy of the ACT. These findings demonstrate the importance of following a standardized protocol to enable the comparison of therapeutic efficacy results across sites and over time.

There are very few studies on therapeutic efficacy and drug resistance in Togo. In a literature review, only one publication on the therapeutic efficacy of ACT in Togo was found [11]. Among 80 patients, 22 were seen on day 3, of whom 20 had cleared their parasites. However, the conclusions from the 2007 study of artemether-lumefantrine are limited, since treatment was not supervised and there was limited follow-up; parents were instructed to return only if their child’s condition persisted after 72 hours. Further, therapeutic efficacy is normally targeted among children under five years of age, and this study was conducted on children aged five years and over.

## Conclusions

Artemether-lumefantrine and artesunate-amodiaquine are both first-line medicines for the treatment of uncomplicated malaria in Togo, and both have shown high efficacy. WHO only recommends a change in treatment policy if the PCR-corrected treatment failure is higher than 10%, which is not the case in Togo. Nevertheless, routine monitoring with adherence to standardized protocols should be continued in order to detect artemisinin resistance if it emerges, and to monitor changes to the therapeutic effect of the partner drug in an ACT.

## Abbreviations

ETF: Early treatment failure; ACPR: Adequate clinical and parasitological response; ACT: Artemisinin-based combination therapy; LCF: Late clinical failure; LPF: Late parasitological failure; NMCP: National Malaria Control Programme; WHO: World Health Organization.

## Competing interests

The authors declare that they have no competing interests.

## Authors’ contributions

MD conceived and designed the study, conducted the research, collected and interpreted the data. YA conducted the research. AB performed the statistical analysis and drafted the manuscript. SK collected the data and conducted the research. HB conducted the research and performed the PCR analysis. YS conceived and designed the study, performed statistical analysis and interpretation. KM conceived and designed the study and conducted the research. All authors read and approved the final manuscript.

## References

[B1] World Health Organization World malaria report 2010 2010 Geneva: World Health Organization

[B2] Sodahlon YK Agbo K Morgah K Adjogble K Avodagbe A Djadou KE Dekou K Pignandi A Kassankogno Y Sukwa T Penali KL Millet P Malvy JM Chloroquine efficacy in the treatment of uncomplicated malaria at three sentinel sites in northern Togo Ann Trop Med Parasitol 2003 97 775 782 10.1179/000349803225002471 14754489

[B3] World Health Organization Assessment and monitoring of antimalarial drug efficacy for the treatment of uncomplicated falciparum malaria 2003 Geneva: World Health Organization

[B4] World Health Organization Methods for surveillance of antimalarial drug efficacy 2009 Geneva: World Health Organization

[B5] World Health Organization Susceptibility of plasmodium falciparum to antimalarial drugs: report on global monitoring 1996-2004 2005 Geneva: World Health Organization

[B6] World Health Organization Methods and techniques for clinical trials on antimalarial drug efficacy: genotyping to identify parasites population 2007 Geneva: World Health Organization

[B7] Premji ZG Abdulla S Ogutu B Ndong A Falade CO Sagara I Mulure N Nwaiwu O Kokwaro G The content of African diets is adequate to achieve optimal efficacy with fixed-dose artemether-lumefantrine: a review of the evidence Malar J 2008 7 244 10.1186/1475-2875-7-244 19032767 PMC2611997

[B8] World Health Organization Global report on antimalarial drug efficacy and drug resistance (2000-2010) 2010 Geneva: World Health Organization

[B9] Owusu-Agyei S Asante KP Owusu R Adjuik M Amenga-Etego S Dosoo DK Gyapong J Greenwood B Chandramohan D An open label, randomized trial of artesunate + amodiaquine, artesunate + chlorproguanil + Dapsone and Artemether + lumefantrine for the treatment of uncomplicated malaria PLoS One 2008 3 e2530 10.1371/journal.pone.0002530 18575626 PMC2430614

[B10] Zwang J Olliaro P Barennes H Bonnet M Brasseur P Bukirwa H Cohuet S D’Alessandro U Djimdé A Karema C Guthmann JP Hamour S Ndiaye JL Mårtensson A Rwagacondo C Sagara I Same-Ekobo A Sirima SB van den Broek I Yeka A Taylor WR Dorsey G Randrianarivelojosia M Efficacy of artesunate−amodiaquine for treating uncomplicated falciparum malaria in sub-Saharan Africa: a multi-centre analysis Malar J 2009 8 203 10.1186/1475-2875-8-203 19698172 PMC2745424

[B11] Djadou KE Agbodjan-Djossou A Azoumah KD Djadou D Lawson-Evi K Balaka B Komlangan A [Artemether-lumefantrine, treatment of child more than 5years old uncomplicated malaria in Tsevie’s hospital, Togo] [in French] Arch Pediatr 2007 14 1463 1464 10.1016/j.arcped.2007.10.003 17962001

